# Porphyrin-engineered nanoscale metal-organic frameworks: enhancing photodynamic therapy and ferroptosis in oncology

**DOI:** 10.3389/fphar.2024.1481168

**Published:** 2024-10-23

**Authors:** Yutao Zou, Jiayi Chen, Xuanxuan Luo, Yijie Qu, Mengjiao Zhou, Rui Xia, Weiqi Wang, Xiaohua Zheng

**Affiliations:** ^1^ The People’s Hospital of Danyang, Affiliated Danyang Hospital of Nantong University, Danyang, Jiangsu, China; ^2^ School of Pharmacy, Nantong University, Nantong, Jiangsu, China; ^3^ School of Public Health, Nantong University, Nantong, Jiangsu, China

**Keywords:** porphyrin, metal-organic framework, photodynamic therapy, ferroptosis, reactive oxygen species

## Abstract

Photodynamic therapy and ferroptosis induction have risen as vanguard oncological interventions, distinguished by their precision and ability to target vulnerabilities in cancer cells. Photodynamic therapy’s non-invasive profile and selective cytotoxicity complement ferroptosis’ unique mode of action, which exploits iron-dependent lipid peroxidation, offering a pathway to overcome chemoresistance with lower systemic impact. The synergism between photodynamic therapy and ferroptosis is underscored by the depletion of glutathione and glutathione peroxidase four inhibitions by photodynamic therapy-induced reactive oxygen species, amplifying lipid peroxidation and enhancing ferroptotic cell death. This synergy presents an opportunity to refine cancer treatment by modulating redox homeostasis. Porphyrin-based nanoscale metal-organic frameworks have unique hybrid structures and exceptional properties. These frameworks can serve as a platform for integrating photodynamic therapy and ferroptosis through carefully designed structures and functions. These nanostructures can be engineered to deliver multiple therapeutic modalities simultaneously, marking a pivotal advance in multimodal cancer therapy. This review synthesizes recent progress in porphyrin-modified nanoscale metal-organic frameworks for combined photodynamic therapy and ferroptosis, delineating the mechanisms that underlie their synergistic effects in a multimodal context. It underscores the potential of porphyrin-based nanoscale metal-organic frameworks as advanced nanocarriers in oncology, propelling the field toward more efficacious and tailored cancer treatments.

## 1 Introduction

Formed by metal nodes and organic linkers, nanoscale metal-organic frameworks (nMOFs) are a subclass of coordination polymers. They exhibit remarkable potential in biomedicine, especially in drug delivery systems (He et al., 2015; Wang W. et al., 2022; Liu et al., 2023). Their high specific surface area and tunable pore structures facilitate the loading of diverse therapeutic agents (Dou et al., 2021), from small molecules to proteins and nucleic acids (Lu et al., 2018; Su et al., 2022; Liu et al., 2023). The chemical stability and biocompatibility of nMOFs ensure safe *in vivo* drug delivery, while surface functionalization enables targeted delivery to specific lesions, optimizing efficacy and minimizing systemic side effects (Wang W. et al., 2022; Wang and Zhang, 2024). These intelligent materials respond to stimuli such as pH, temperature, or light, achieving controlled drug release and enhancing therapeutic precision and efficiency (Yang J. et al., 2023; Zhou S. et al., 2024). Porphyrin-based nMOFs, which are particularly effective in phototherapy, have garnered significant interest (Lan et al., 2018; He et al., 2019; Zhang D. et al., 2019; Xu et al., 2023).

Photodynamic therapy (PDT) is an innovative approach to cancer treatment (Shen et al., 2022). It leverages the selectivity and low invasiveness of light-activated photosensitizers (PSs) (Lucky et al., 2015; Gunaydin et al., 2021). Concentrated within tumors and activated by specific wavelengths, PDT precisely targets malignant cells while sparing surrounding healthy tissue (Lan et al., 2019). Its non-invasive nature reduces patient discomfort and recovery time compared to surgery and radiation (Chatterjee et al., 2008). The repeatability of PDT allows for repeated treatments without escalating side effects, offering sustained therapeutic options for recurrent or residual tumors (Kwiatkowski et al., 2018). Additionally, PDT demonstrates synergistic potential with other cancer treatments like chemotherapy and immunotherapy, potentially overcoming resistance and enhancing overall outcomes (Wang et al., 2019; Jiang et al., 2023a; Zhou S. et al., 2024).

Ferroptosis is an emerging cancer therapy (Zhao et al., 2019; Wang Y. et al., 2022). It induces iron-dependent lipid peroxidation, which disrupts cellular membranes and triggers non-apoptotic, iron-mediated cell death (Chen et al., 2021; Jiang et al., 2021; Li et al., 2022). Its specificity for cancer cells with dysregulated iron metabolism or antioxidant imbalance provides precise elimination with minimal impact on normal cells (Cheng et al., 2022; Lei et al., 2022). Ferroptosis is effective against drug-resistant tumors. Theoretically, its low toxicity suggests it may cause fewer systemic side effects, thereby improving quality of life (Li et al., 2020; Nie et al., 2022). Combined with existing therapies, ferroptosis amplifies treatment effectiveness, thereby offering comprehensive treatment regimens (Wang et al., 2023a; Chang et al., 2024).

The synergy between PDT and ferroptosis is striking. PDT-generated reactive oxygen species directly assault tumor cells and accelerate lipid peroxidation. This promotion of ferroptosis doubly compromises tumor survival (Chen et al., 2023a; Cai X. et al., 2024). Ferroptosis-generated peroxides and molecular oxygen mitigate hypoxic tumor environments, enhancing PDT’s efficacy under hypoxic conditions (Fang et al., 2024; Huang et al., 2024; Wang Y. et al., 2024). Combined strategies that circumvent resistance issues associated with monotherapies broaden treatment horizons (Wei et al., 2022; Xiong et al., 2024; Yang et al., 2024).

Employing nMOFs as carriers for porphyrin-based photosensitizers, such as Fe-TCPP nMOFs, ensures the effective implementation of PDT. These nMOFs also orchestrate iron ion release, which induces ferroptosis in tumor cells. This approach establishes a synergistic assault strategy (Cai M. et al., 2024). The utilization of porphyrin-based nMOFs for tumor PDT and ferroptosis therapy is endowed with several advantages: 1) The high porosity and large surface area of nMOFs enable a high loading capacity of PSs, facilitating the generation of abundant reactive oxygen species (ROS) (Ni et al., 2022; Liu et al., 2023). This leads to the depletion of glutathione (GSH), an increase in lipid peroxidation, and the induction of ferroptosis. 2) NMOFs exhibit excellent biocompatibility and degradability. When they degrade, they release Fe^3+^ that can be converted into Fe^2+^, triggering the Fenton reaction to produce oxygen (Pan et al., 2022). This oxygenation counteracts the hypoxic microenvironment, thereby enhancing the efficacy of PDT. 3) The facile surface modification of nMOFs allows for the introduction of targeting moieties such as hyaluronic acid (HA) or cell membranes. This enables precise delivery to specific tumor types while minimizing interference with healthy tissues (Yuan et al., 2024). 4) The porous structure of nMOFs confers multifunctionality, permitting the co-delivery of chemotherapeutics, gas-releasing agents, or immunostimulants. This integration of PDT, ferroptosis, and additional therapies can create synergistic effects and optimize outcomes with reduced side effects (Chen et al., 2023b; Yang F. et al., 2023). 5) PDT exhibits remarkable potential for immune modulation and can stimulate the host’s immune response against distant and metastatic tumors. This capability significantly overcomes the spatial limitations inherent to strategies for inducing ferroptosis. Precise regulation of PDT enables effective control over widely dispersed tumors (Castano et al., 2006). 6) NMOFs based on porphyrins, such as complexes formed by Fe^3+^ and tetracarboxyporphyrin; offer a straightforward approach to synergizing PDT with ferroptosis. These unique formation processes of nMOFs provide an efficient method for this integration (Yang F. et al., 2023). This streamlined design, without complex molecular architecture, can demonstrate potent antitumor capabilities.

**FIGURE 1:**
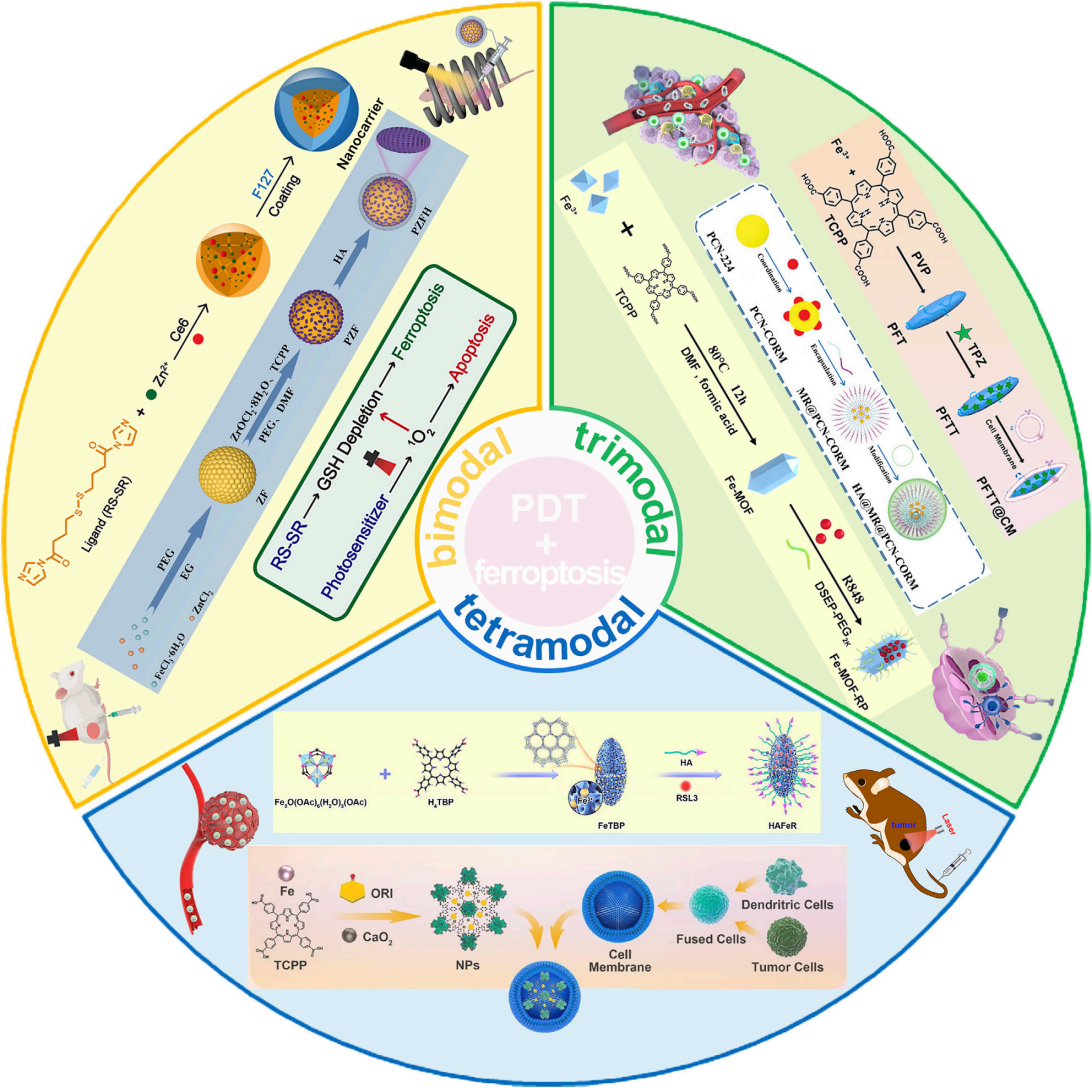
Schematic illustration depicting the utilization of porphyrin-based nMOFs in the synergistic treatment of tumors through PDT and ferroptosis induction, complemented by additional therapeutic modalities. Reproduced with permission from Meng et al. (2019) and Fan et al. (2024). Copyright (2019, 2024), American Chemical Society. Reproduced with permission from Yang F. et al. (2023) and Yuan et al. (2024). Copyright (2023, 2024), John Wiley & Sons, Inc. Reproduced with permission from Pan et al. (2022), Chen et al. (2023b), Cai M. et al. (2024). Copyright (2022, 2023, 2024), Elsevier.

This review is dedicated to comprehensively dissecting the cutting-edge advancements in employing porphyrin-based nMOFs for the synergistic treatment of tumors through PDT and ferroptosis (Figure 1). We delve into the design rationale of nMOFs, with a particular emphasis on how porphyrin nMOFs catalyze the production of enhanced levels of ROS, thereby inducing oxidative stress. This dual action promotes apoptosis while simultaneously depleting GSH and indirectly inhibiting glutathione peroxidase 4 (GPX4), culminating in the activation of ferroptosis-a distinctive pathway pivotal for the innovation and refinement of oncological treatment strategies. Moreover, this review systematically consolidates recent studies leveraging the post-modification versatility of nMOFs (Figure 1). By integrating chemodynamic therapy (CDT), conventional chemotherapy, and immunotherapy, these studies aim to augment the effectiveness of PDT and ferroptosis treatments. Innovative methodologies open novel avenues for addressing clinical challenges posed by hypoxic, drug-resistant, and highly metastatic tumors. Such approaches highlight the vast potential of nMOFs as a multifaceted platform in cancer therapeutics.

## 2 NMOFs for PDT and ferroptosis

As a vanguard approach in cancer treatment, PDT modality hinges on the induction of apoptosis by activating the intrinsic cell suicide program to eradicate malignant cells (Qi et al., 2022; Ma et al., 2024). Recent insights have shown that singlet oxygen (^1^O_2_), a highly reactive species generated during PDT, can propel apoptotic pathways. Additionally, it depletes intracellular GSH. This depletion catalyzes ferroptosis, an atypical mode of cell death (Sun et al., 2022; Wei et al., 2022; Xiong et al., 2024).

**FIGURE 2:**
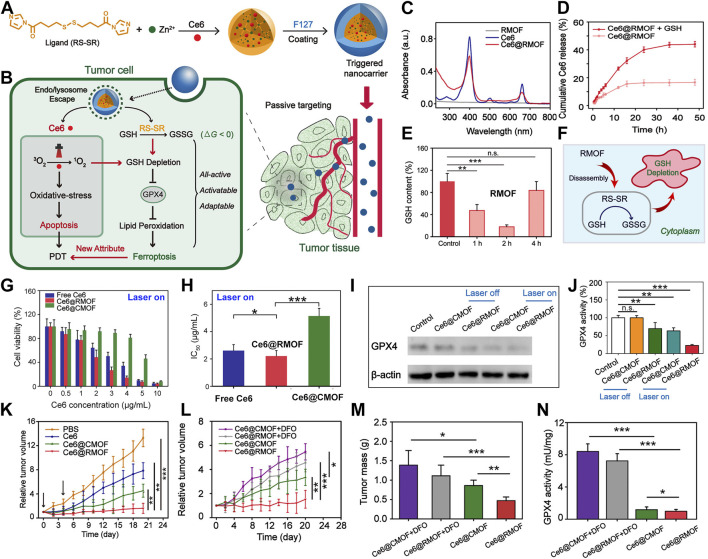
**(A)** Conceptual depiction of all-active MOF nanocarriers for **(B)** dual apoptosis and ferroptosis antitumor PDT. **(C)** UV-vis spectra of RMOF, free Ce6, and Ce6@RMOF (20 μM Ce6). **(D)** Ce6@RMOF stability ± 10 mM GSH (n = 3). **(E)** Redox-responsive MOF (RMOF) nanocarrier; untreated cells as control. **(F)** Thiol-disulfide exchange mechanism leading to GSH depletion. **(G)** Cell viability post-treatment with Ce6, Ce6@CMOF, or Ce6@RMOF + light (660 nm, 100 mW/cm^2^, 10 min) (n = 5). **(H)** IC50 values of three treatments under laser. **(I)** GPX4 expression in 4T1 cells treated with Ce6-loaded CMOF/RMOF ± laser (660 nm, 100 mW/cm^2^, 5 min). **(J)** GPX4 activity post-incubation with Ce6@CMOF/RMOF. **(K)** Tumor growth post-four treatments (PBS, Ce6, Ce6@CMOF, Ce6@RMOF). **(L)** Growth curves for mice treated with Ce6@CMOF/RMOF (5 mg/kg Ce6) ± DFO. **(M)** Tumor mass quantification on day 20. **(N)** Iron chelation impact on GPX4 activity in tumors. (n.s., not significant, *p < 0.05, **p < 0.01, ***p < 0.001). Reproduced with permission from Meng et al. (2019). Copyright (2019), American Chemical Society.

Through the employment of redox-responsive nanocarriers, this cascade can be meticulously modulated (Chen et al., 2023c; Wang et al., 2023c; Zhou C. et al., 2024). Meng et al. (2019) introduced an innovative strategy involving the construction of a high-performance MOF nanoparticle (NP) system using imidazole ligands containing disulfide bonds conjugated with zinc ions, effectively loading the photosensitizer chlorin e6 (Ce6) (Figure 2A). These Ce6-loaded MOF NPs can trigger substantial depletion of GSH within the 4T1 murine breast cancer cell line via a chemical exchange reaction between disulfide bonds and intracellular thiol groups, even under non-illuminated conditions. The marked reduction in GSH levels leads to the inactivation of GPX4, a pivotal enzyme for maintaining cellular redox homeostasis, which exacerbates cytotoxicity and promotes cell death (Figure 2B). UV spectra confirm the successful encapsulation of Ce6 (Figure 2C), and release studies indicate that Ce6 is efficiently liberated in the presence of GSH (Figure 2D), showcasing the redox-responsiveness of the nanomaterial and its contribution to enhanced PDT efficacy. GSH assays demonstrate that the redox-responsive MOF (RMOF) nanocarrier significantly diminishes GSH levels in 4T1 cells (Figure 2E), an effect attributed to thiol-disulfide exchange (Figure 2F). GSH levels rebound after 4 h, possibly due to the cell’s initiation of GSH replenishment synthesis. At low doses, both RMOF and CMOF (control MOF nanocarriers) have limited impact on cell viability; irrespective of illumination (Figure 2G). However, at higher doses, RMOF exhibits superior cytotoxicity, regardless of light exposure (Figure 2G). Specifically, upon irradiation, the IC50 value of Ce6@RMOF is notably lower than that of Ce6@CMOF (*p* < 0.001) (Figure 2H), owing to the enhancement of efficacy from GSH depletion and rapid drug release (Figure 2H).

GSH serves as a critical regulator of apoptosis and ferroptosis, facilitating detoxification of lipid peroxides through GPX4. The Ce6@RMOF nanocarrier employs dual pathways to deplete GSH and induce ferroptosis (Figure 2B): thiol-disulfide exchange and Ce6-mediated singlet oxygen-induced thiol oxidation. In contrast, Ce6@CMOF relies solely on singlet oxygen. Protein expression analysis of GPX4 corroborates this theory (Figure 2I). GPX4 activity assays reveal that Ce6@RMOF initiates ferroptosis in the absence of light, unlike Ce6@CMOF (Figure 2J). Light exposure amplifies the suppression of GPX4, with Ce6@RMOF demonstrating exceptional performance. In a 4T1 tumor-bearing mouse model, the MOF carriers markedly curtail tumor progression (Figure 2K), underscoring their clinical potential. Co-administration of deferoxamine attenuates the antitumor activity of both Ce6@CMOF and Ce6@RMOF (Figures 2L, M), highlighting the role of ferroptosis in PDT. Assays measuring GPX4 activity affirm the contribution of ferroptosis to antitumor PDT (Figure 2N).

These findings furnish compelling evidence, offering new perspectives for the multidimensional development of PDT therapies. They suggest that by orchestrating the interplay between ferroptosis and apoptotic pathways induced by PDT, one can achieve more efficacious eradication of tumor cells, heralding novel avenues for future cancer treatment strategies.

## 3 NMOFs for PDT and ferroptosis (magneto-optical co-stimulation)

By incorporating disulfide bonds and leveraging the ROS generated by PDT, it is possible to reduce intracellular GSH levels, indirectly affecting the activity of GPX4, thereby instigating the signaling pathway for ferroptosis (Liu et al., 2022; Zhou C. et al., 2024). This leads some researchers to hypothesize that by employing diverse triggering mechanisms to impose greater oxidative stress; the more accumulation of LPO may be precipitated for further amplifying the ferroptotic process.

Optical and magnetic technologies are increasingly showing their exceptional potential in oncology. These technologies are inherently non-invasive. They do not involve radioactivity. Additionally, they enable remote and immediate responses. Precise targeting is another key feature that enhances their effectiveness (Ouyang et al., 2022; Zhang et al., 2023). These approaches not only circumvent the adverse effects associated with conventional treatments, such as tissue damage and radiation hazards, but also offer a more refined level of spatiotemporal control, thus making on-demand activation of therapeutic mechanisms feasible (Thorat et al., 2019; Yuan et al., 2024). The synergy of optical and magnetic methodologies allows for precise intervention in tumor tissues, which promise a more personalized and effective strategy for tumor ablation (Ouyang et al., 2022; Yuan et al., 2024). Grounded in these principles, Li et al. innovatively combined optical and magnetic techniques. They dramatically augmented the intrinsic peroxidase-like activity of iron-based core-shell MOFs. By capitalizing on singlet oxygen production from PDT, they cultivated an intensified oxidative stress environment (Yuan et al., 2024). This strategy remarkably enhanced the tumor-suppressive effects when PDT and ferroptosis act in concert.

**FIGURE 3:**
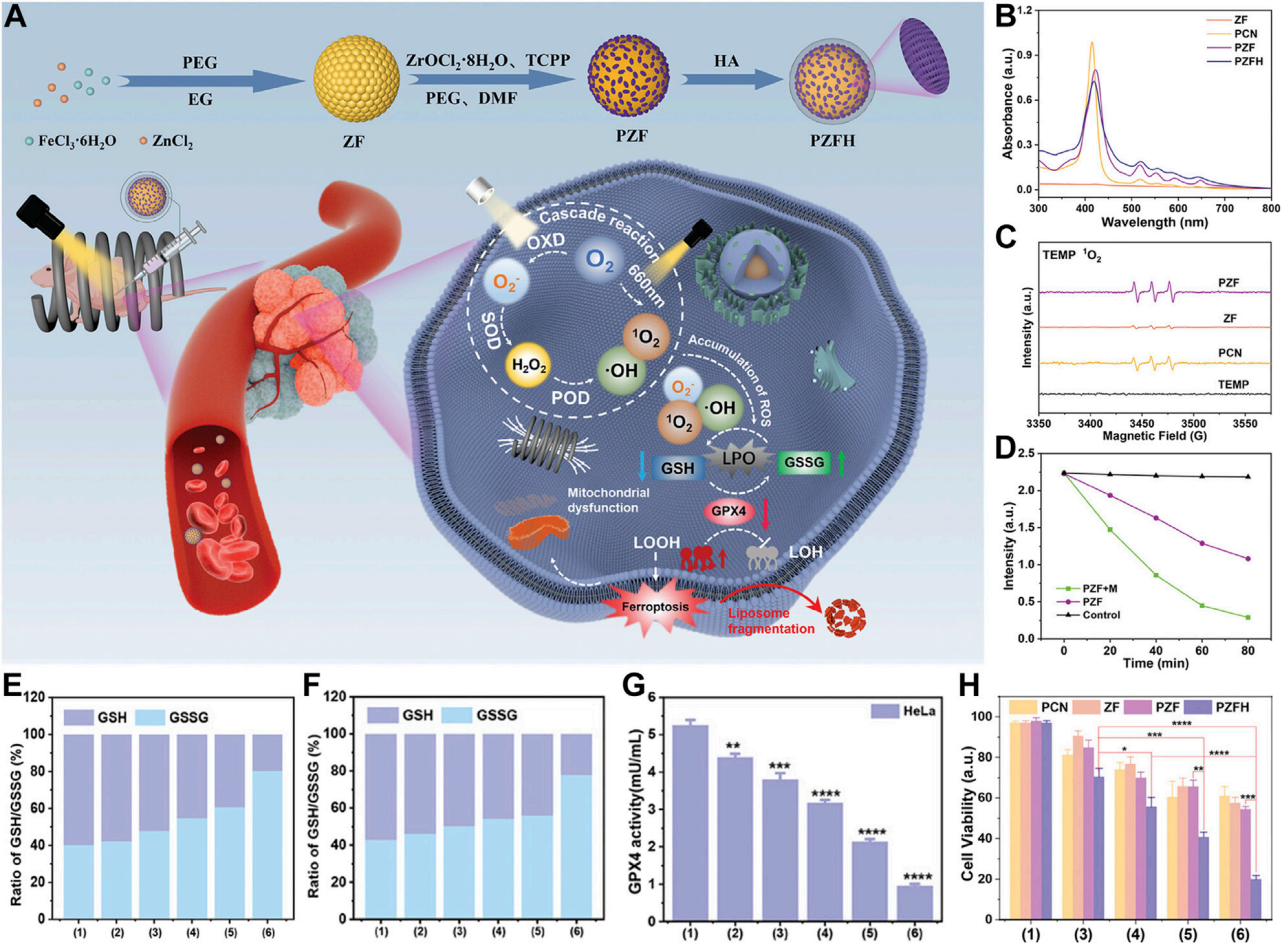
**(A)** Diagram of PCN@ZF-HA (PZFH) synthesis and magnetophotonic tumor ferroptosis via enzymatic cascade. **(B)** UV-vis spectral profile. **(C)** ESR spectrum with TEMP adducts. **(D)** GSH absorbance intensity at 412 nm over time for PZF ± AMF. **(E, F)** GSH/GSSG ratios in HeLa and MCF-7 cells under various treatments. **(G)** GPX4 activity in HeLa cells post-treatment (units: mU/mL). **(H)** Cytotoxicity assessment in HeLa cells under different conditions [Treatments: 1 = Control, 2 = PZFH, 3 = PZFH+Light (Xenon), 4 = PZFH+Light (Xenon, 660 nm), 5 = PZFH+Light (Xenon)+Magnetic Field, 6 = PZFH+Light (Xenon, 660 nm)+Magnetic Field]. Reproduced with permission from Yuan et al. (2024). Copyright (2024), John Wiley & Sons, Inc.

The research team ingeniously designed and synthesized a magnetically and optically dual-controlled multi-level enzyme-mimetic antitumor nanosystem, PCN@ZF-HA (PZFH). The nanosystem seamlessly integrates magnetic zinc ferrite (ZnFe_2_O_4_, abbreviated as ZF) with porphyrin-based zirconium metal-organic frameworks (PCN). The system is functionalized with hyaluronic acid for optimized integration (Figure 3A) (Yuan et al., 2024). HA enhances the targeting capability of the nanomaterials, while the significant magneto-optical responsiveness and ROS generation capacity equip the nanosystem with robust *in vivo* and *in vitro* tumor suppression capabilities (Figure 2A). Material characterization reveals the rationality of the design, with the characteristic absorption peaks of porphyrins and four non-specific absorption peaks confirming the formation of the PCN framework (Figure 3B). Electron spin resonance (ESR) spectroscopy confirms the generation of singlet oxygen by PZFH, attesting to its PDT efficacy (Figure 3C). Under alternating magnetic field (AMF), PZFH accelerates the degradation of GSH, depleting it within 80 min at a doubled rate, indicating magnetic field-enhanced ROS production (Figure 3D). The rise in LPOs indicates ferroptosis, a process that GSH can suppress. With laser and AMF stimulation, the GSH/GSSG ratio drops to 2. This suggests that the nanocomposite facilitates GSH oxidation, which is conducive to LPO accumulation and ferroptosis induction (Figures 3E, F). GPX4 inhibits LPOs, which will alleviate ferroptosis. When PZFH is co-cultured with stimuli, reduced GPX4 activity is observed in HeLa cells. This confirms that the complex downregulates GPX4. The downregulation elevates LPOs and induces ferroptosis (Figure 3G). 3-(4,5-Dimethylthiazol-2-yl)-2,5-diphenyltetrazolium bromide (MTT) is a common reagent used for measuring cell viability (Chen et al., 2020; Zhao et al., 2021). MTT assays validate that PZFH exhibits optimal tumor suppression under combined photo-magnetic stimulation (Figure 3H).

This pioneering nanoplatform leverages the remote activation properties of magnetic materials to enhance the peroxidase activity of iron ions. It also exploits the photosensitization effect of porphyrin structures under light, generating highly reactive ^1^O_2_. This dual-strike mechanism effectively triggers the ferroptotic pathway in tumor cells, heralding a promising avenue for the development of novel anticancer therapeutic modalities.

## 4 nMOFs for PDT + ferroptosis + gas therapy

The synergistic enhancement of ROS generation via combined photo/magnetic effects achieves superior PDT and ferroptotic outcomes. In addition to ROS, current research focusing on carbon monoxide (CO) and nitric oxide (NO) is also a hotspot. NO and CO possesses various biological functions within the organism (Chen et al., 2019; Wu et al., 2019). Additionally, gases like CO and NO can augment the photodynamic sensitivity of porphyrin nMOFs. Such gases have the potential to facilitate ferroptosis (Liu et al., 2021; Zheng Q. et al., 2021; Jiang et al., 2023b).

**FIGURE 4:**
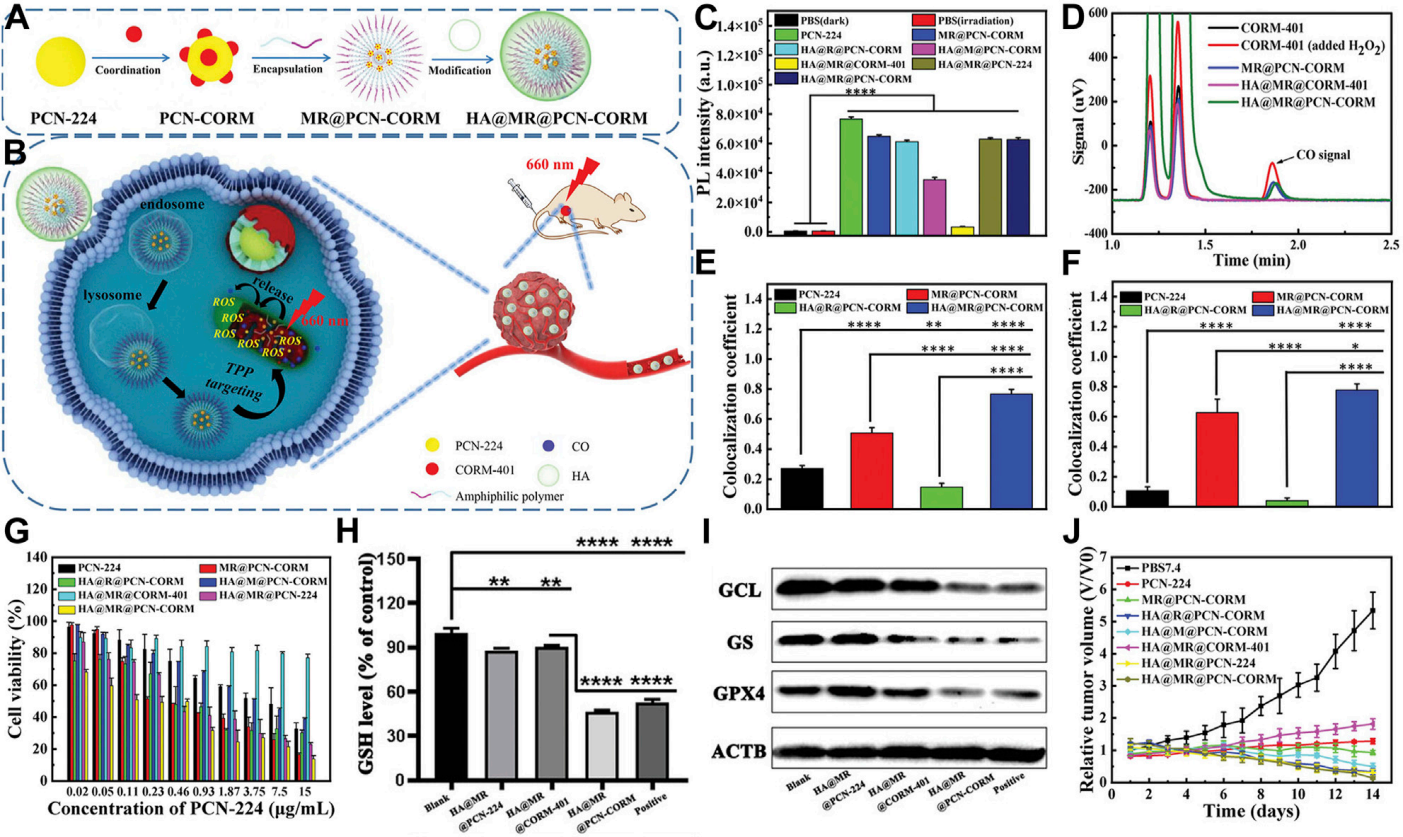
**(A)** Composition overview of HA@MR@PCN-CORM for integrated PDT and gas therapy. **(B)** Synergistic action mechanism of HA@MR@PCN-CORM in combined PDT and gas therapy for tumor treatment. **(C)** ROS assessment across different conditions, monitored by DCFH fluorescence. **(D)** Qualitative CO detection from CORM-401, post-H_2_O_2_ addition, MR@PCN-CORM, HA@MR@CORM-401, and HA@MR@PCN-CORM. **(E)** Image correlation coefficient between PCN-224 and mitochondrial staining. **(F)** Image correlation coefficient between DCF and mitochondrial staining. **(G)** Cytotoxicity evaluation of various formulations on 4T1 cells post-illumination at 660 nm for 30 min. **(H)** Intracellular GSH level determination in 4T1 cells treated with PBS7.4, HA@MR@PCN-224, HA@MR@CORM-401, and Erastin. **(I)** WB analysis of GPX4, GS, and GCL protein expressions in 4T1 cells. **(J)** Tumor weight comparison. Reproduced with permission from Yang F. et al. (2023). Copyright (2023), John Wiley & Sons, Inc.

Peng et al. engineered an innovative amphiphilic polymer incorporating ROS-responsive units and triphenylphosphine end groups. This polymer self-assembles around the porous MOF (PCN-224), which is constructed from tetracarboxyphenylporphyrin coordinated with Zr^4+^. The assembly simultaneously loads the CO-releasing molecule CORM-401. The structure culminates in a hyaluronic acid shell (Figure 4A) (Yang F. et al., 2023). This nanosystem precisely targets mitochondria, crucial in orchestrating both apoptosis and ferroptosis in cancer cells. Near-infrared light activation causes PCN-224 to generate ROS and release CO. These processes accelerate ferroptosis and apoptosis, enhancing antitumor efficacy (Figure 4B). The evaluation of ROS production using DCFH demonstrated that nanoparticles containing thioether (TK) bonds exhibit superior ROS generation efficiency. These TK bonds facilitate the exposure of PCN-CORM to light, affirming the controlled release of CO-modulated ROS. This, in turn, enhances PDT efficacy. Chromatographic analysis confirmed the ROS-triggered release of CO from CORM-401, showcasing the ROS-dependent CO release characteristics of HA@MR@PCN-CORM (Figures 4C, D). Co-localization studies revealed an excellent match between HA@MR@PCN-CORM and the mitochondrial marker MitoTracker in 4T1 cells (Figure 4E), validating the enhancement of mitochondrial delivery by HA and triphenylphosphine (TPP). DCFH assays disclosed ROS generation in mitochondria upon irradiation (Figure 4F).

HA@MR@PCN-CORM showed maximal toxicity toward 4T1 cells under light exposure in cytotoxicity assays. This effect is due to the dual-targeting of HA and TPP, the enhanced photosensitivity from TK bonds, and the release of CO from mitochondria induced by ROS (Figure 4G). HA@MR@PCN-CORM influenced ferroptosis signaling, including GSH depletion and GPX4 inactivation. Western blot analysis revealed downregulation of GCL and GS expression, promoting ferroptosis and apoptosis, with the lowest enzyme and GPX4 expression observed in the HA@MR@PCN-CORM group (Figures 4H, I). *In vivo* experiments demonstrated that HA@MR@PCN-CORM delivered optimal PDT-mediated antitumor efficacy in BALB/c mice bearing 4T1 cell xenografts (Figure 4J).

In a cascade of meticulously orchestrated biochemical reactions, the precise intramitochondrial release of CO within tumor cells inhibits GSH synthesis. This inhibition adroitly precipitates the onset of ferroptosis while concurrently hastening apoptosis. The result is a potent antitumor synergistic effect. This innovative delivery system not only underscores the novel potential of PDT in oncology but also paves the way for the development of more efficacious and targeted cancer therapeutic strategies.

## 5 nMOFs for PDT + ferroptosis + chemotherapy

Capitalizing on the unique characteristics of the tumor microenvironment, researchers have engineered a nanodrug delivery system capable of harnessing a series of cascading reactions to achieve efficient cancer therapy (Battistella et al., 2021; De Lázaro and Mooney, 2021; Pang et al., 2024). By exploiting the hypoxic and redox conditions within tumors, some approaches activate the drug payload. This activation enables a precise strike against cancerous tissues. Integrating PDT, ferroptosis, and chemotherapy in a synergistic manner enhances the effectiveness of such treatments (Pan et al., 2023; Zhu et al., 2023; Wu X. et al., 2024).

**FIGURE 5:**
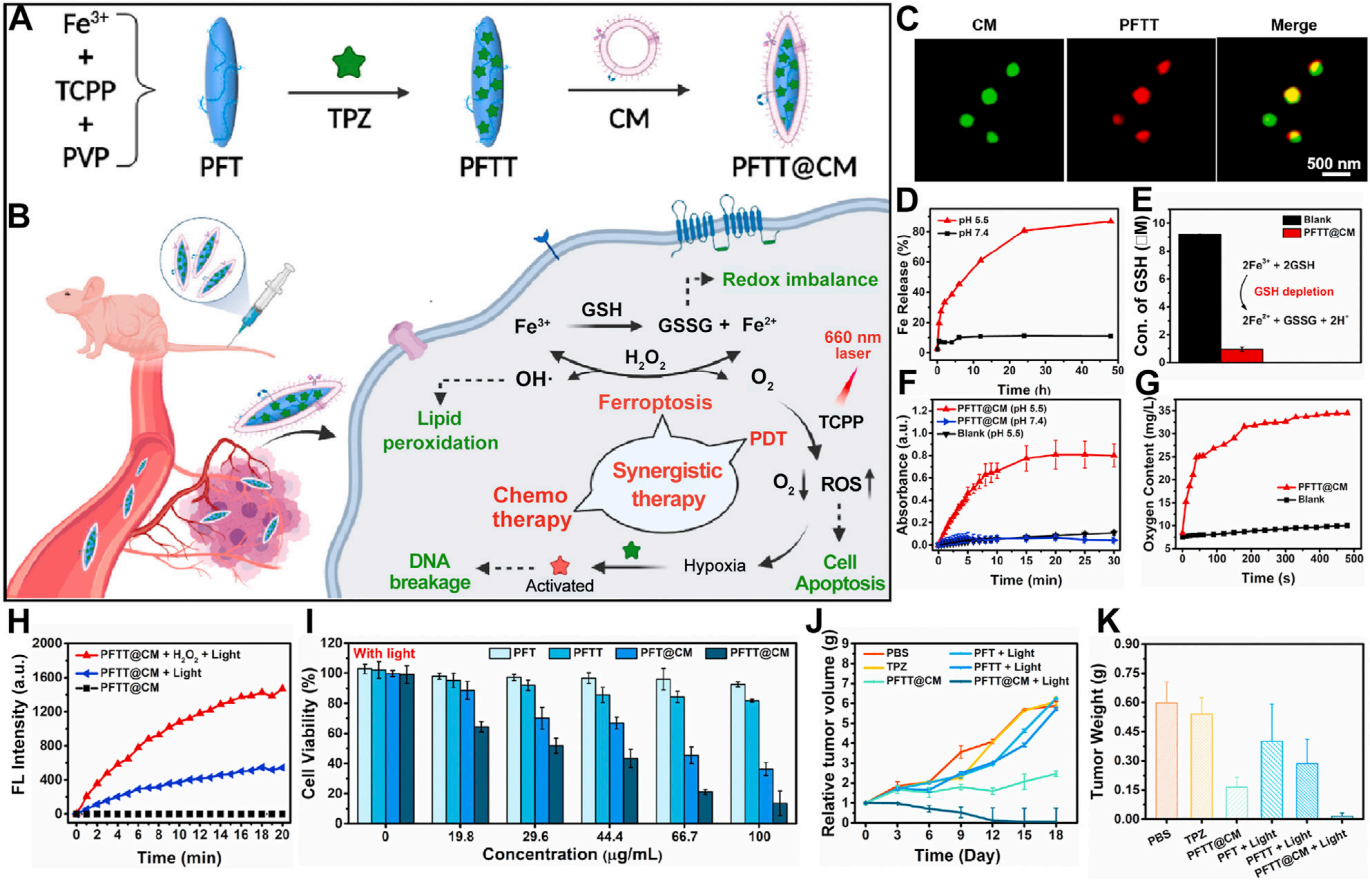
**(A)** Preparation scheme for cancer-cell-membrane-cloaked PFTT@CM nanocomposites. **(B)** Combined therapeutic efficacy of PFTT@CM, integrating ferroptosis, PDT, and hypoxia-targeted chemotherapy. **(C)** Super-resolution imaging of PKH67 (green)-stained PFTT@CM highlighting cell membrane features. **(D)** pH-dependent Fe^3+^ release kinetics from PFTT@CM at physiological (pH 7.4) and acidic (pH 5.5) conditions. **(E)** GSH depletion efficacy of PFTT@CM facilitated by Fe^3+^ ions. **(F)** Continuous H_2_O_2_ catalysis by PFTT@CM, assessed via TMB assay over 30 min. **(G)** Oxygen evolution kinetics from PFTT@CM in H_2_O_2_ solutions over time. **(H)** SOSG fluorescence dynamics in the presence of PFTT@CM + H_2_O_2_ + light, PFTT@CM + light, or PFTT@CM alone. **(I)** Light-dependent cytotoxicities of PFT, PFTT, PFT@CM, and PFTT@CM against MDA-MB-231 cells, quantified by CCK8 assay. **(J)** Comparative tumor growth trajectories. **(K)** Final tumor weights changes. Reproduced with permission from Pan et al. (2022). Copyright (2022), Elsevier.

Chen et al. developed an innovative nanocarrier, designated PFTT@CM, composed of iron-tetra(4-carboxyphenyl)porphyrin (Fe-TCPP) NMOFs encapsulating tirapazamine (TPZ) and camouflaged with cancer cell membranes, ensuring tumor-specific delivery (Figure 5A) (Pan et al., 2022). This system leverages homologous membrane masking to evade immune surveillance and concentrate in tumor regions. Following cancer cell uptake, PFTT@CM is activated through Fenton reactions and redox processes. This activation depletes GSH stores and generates hydroxyl radicals (•OH) and oxygen. As a result, ferroptosis is triggered, amplifying the efficacy of PDT. Activated under hypoxic conditions, radicals produced by TPZ have a strong cytotoxic effect (Figure 5B). Super-resolution microscopy images confirm the successful co-localization of PFTT with cancer cell membranes, indicating effective membrane encapsulation (Figure 5C). The nanocarrier efficiently releases Fe^3+^ under acidic conditions. This accelerates the conversion of GSH to GSSG. The compromise of cellular antioxidant defenses instigates ferroptosis (Figure 5D). Interaction of Fe^3+^ with GSH yields Fe^2+^, which catalyzes hydrogen peroxide (H_2_O_2_) decomposition under acidic conditions via the Fenton reaction, generating oxygen and •OH (Figure 5E). Experimental data validate the significantly enhanced generation of •OH by PFTT@CM at pH 5.5 (Figure 5F), alongside a marked increase in O_2_ concentration (Figure 5G). The elevated O_2_ concentration could promote PDT. Utilizing the SOSG probe, the researcher demonstrate that PFTT@CM markedly elevates ^1^O_2_ generation under light exposure, with enhanced effects in the presence of H_2_O_2_ (Figure 5H). Comparative studies reveal that PFT@CM, without TPZ, exhibits light-dependent cytotoxicity. This cytotoxicity is concentration-related. The findings confirm the combined effect of ferroptosis and PDT. PFTT@CM displays the strongest antitumor activity under illumination, owing to the synergistic action of ferroptosis, PDT, and TPZ chemotherapy (Figure 5I). In tumor-bearing mouse models, PFTT@CM combined with light irradiation significantly inhibits tumor growth. This treatment also reduces tumor weight compared to other treatment groups. These results underscore the importance of targeted delivery and synergistic therapy in cancer management (Figures 5J, K).

This therapeutic paradigm adeptly modulates the TME and employs a precisely choreographed cascade of synergistic mechanisms to achieve optimized therapeutic outcomes. This integrated multi-functional platform highlights the substantial potential of porphyrin-based nMOFs in synergistic PDT and ferroptosis treatment strategies. It also heralds a promising future for bioinspired recognition technologies. Additionally, it points towards the potential of TME-adaptive approaches in targeted combination therapies. Due to its target specificity and nuanced regulation of the TME, this combinatorial treatment modality significantly enhances antitumor efficacy. It also charts new courses for future cancer therapeutics.

## 6 nMOFs for PDT + ferroptosis + immunotherapy

For an extended period, three major challenges have plagued the field of oncology: the hypoxic state within tumors, multidrug resistance of cancer cells, and the metastatic propensity of certain malignancies (Brown and Wilson, 2004; Kapse-Mistry et al., 2014; Bergers and Fendt, 2021). While employing hypoxia-targeted chemotherapeutics such as TPZ can amplify the therapeutic effects of porphyrin nMOFs in PDT and ferroptosis treatments, existing strategies fall short in managing tumors prone to distant metastasis (Liu et al., 2018; Ye et al., 2022; Jiang et al., 2023b). Against this backdrop, immunotherapy-integrated treatment modalities have emerged (Zhang B. et al., 2019; Gao et al., 2020; Hu et al., 2020). These modalities aim to reinforce the synergy between PDT, ferroptosis therapy, and the host’s immune system. This reinforcement aims to achieve more pronounced suppression of metastatic tumors (Lan et al., 2018; Song et al., 2018; Hua et al., 2021). Such a comprehensive treatment approach addresses critical issues in cancer therapy. It may also precipitate a paradigm shift in clinical practice. This shift offers patients more holistic and efficacious treatment regimens (Warszyńska et al., 2023).

**FIGURE 6:**
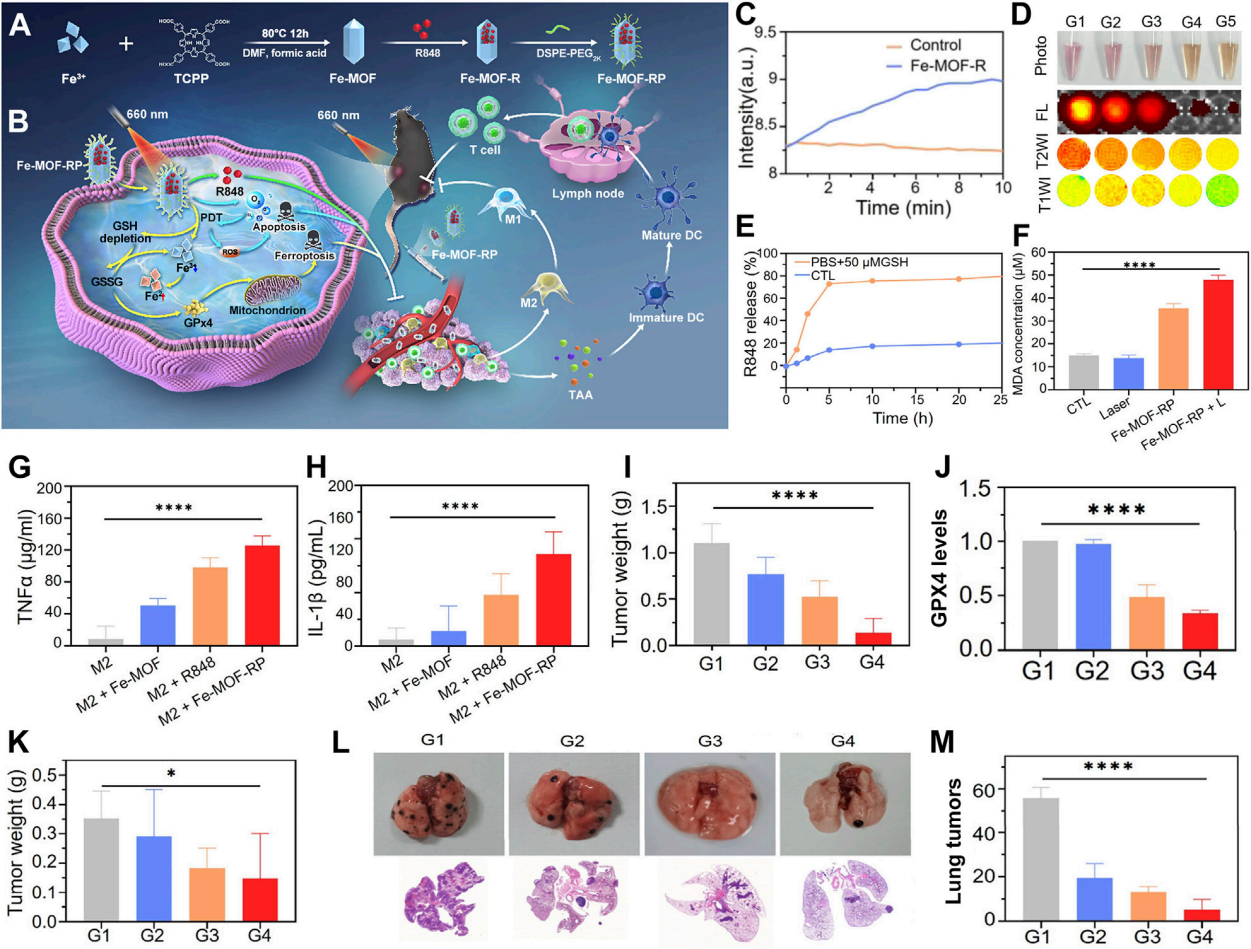
**(A)** Synthesis strategy for the Fe-TCPP-R848-PEG (Fe-MOF-RP) nanozymes. **(B)** Action mechanism of light-triggered nanozymes in tumor therapy. **(C)** Catalytic efficiency of Fe-MOF-R in hydrogen peroxide decomposition. **(D)** Response of Fe-TCPP MOFs to GSH, evaluated by photographic, fluorescence, and MRI imaging. **(E)** Kinetic profile of R848 release in GSH buffer solution. **(F)** Comparative analysis of MDA levels across treatment groups. **(G)** Influence of Fe-MOF-RP on TNFα expression in macrophages, quantified by ELISA. **(H)** Impact of Fe-MOF-RP on IL-1β expression in macrophages, assessed via ELISA. **(I)** Changes in tumor weight post-treatment. **(J)** Quantification of GPX4 protein expression levels across panels G1-G4: G1-Control, G2-R848, G3-Fe-MOF-RP, G4-Fe-MOF-RP + Laser. **(K)** Statistical analysis of distal tumor weights (sample size n = 8). **(L)** Observation of lung metastasis effects from various treatment groups in organ isolates and histological sections. **(M)** Enumeration of lung tumor nodules. Reproduced with permission from Fan et al. (2024). Copyright (2024), American Chemical Society.

For instance, Liu et al. innovatively developed a light-activated nanozyme, Fe-TCPP-R848-PEG (FeMOF-RP), which can be used to remodel the immunosuppressive tumor microenvironment (Figure 6A) (Fan et al., 2024). The Fe-TCPP MOF serves not only as a pivotal catalytic component to combat tumors but also as a biocompatible carrier to enhance the delivery efficiency of immunostimulants, optimizing circulation time and tumor accumulation. Concurrently, it catalyzes the decomposition of intratumoral hydrogen peroxide into oxygen, thus augmenting the efficacy of PDT. The synergistic action of ferroptosis and PDT, which promotes the release of tumor-associated antigens, triggers immunogenic cell death. Light-controlled release of R848 stimulates dendritic cell maturation. It also transforms pro-tumorigenic M2 macrophages into anti-tumorigenic M1 phenotypes. This transformation reshapes the tumor immune landscape (Figure 6B).

The peroxidase activity of Fe-MOF was substantiated through portable dissolved oxygen meter monitoring. This monitoring demonstrated its capacity to catalyze hydrogen peroxide into oxygen. This catalysis effectively alleviates tumor hypoxia (Figure 6C). The responsiveness of Fe-MOF-RP to GSH manifests in decreased fluorescence signals and altered magnetic resonance signal intensity due to GSH consumption, alongside GSH-facilitated controlled release of R848 (Figures 6D, E). The ferroptosis-inducing capability of Fe-MOF-RP was confirmed through lipid peroxidation experiments. Treatment with Fe-MOF-RP notably increased lipid peroxidation. This increase was further exacerbated by laser irradiation (Figure 6F). Fe-MOF-RP modulated macrophage secretion of IL-1β and TNF-α, with particularly significant effects observed in R848, Fe-TCPP MOFs, and Fe-MOF-RP treatments, where Fe-MOF-RP demonstrated the most pronounced impact (Figures 6G, H). The therapeutic efficacy of Fe-MOF-RP on established tumors was evident. The best inhibition was achieved when the treatment was combined with laser therapy. This combination was accompanied by downregulation of GPX4 expression (Figures 6I, J). In bilateral tumor models, Fe-MOF-RP in conjunction with laser therapy exhibited notable suppression of distal tumor growth (Figure 6K). Fe-MOF-RP coupled with laser treatment showed the strongest inhibition of lung metastases. This approach promoted immune cell infiltration. It also effectively controlled tumor growth and prevented pulmonary metastasis (Figures 6L, M).

This pioneering therapeutic formulation establishes a seamless integration among PDT, ferroptosis therapy, and immunotherapy. This integration forms a sophisticated synergistic network. Compared to conventional PDT, this system exhibits distinctive advantages. It efficiently converts hydrogen peroxide to oxygen. This conversion ingeniously overcomes the oxygen supply bottleneck often encountered during treatment. As a result, it ensures the full deployment of photodynamic effects. Moreover, this nanosystem capitalizes on the tumor microenvironment’s GSH reservoir. It releases ferrous ions to induce ferroptosis. This mechanism not only directly assaults cancer cells but also triggers a robust antitumor immune response, thus significantly amplifying therapeutic outcomes. In practical application, this therapy effectively restrains primary tumor growth. It also exerts a significant suppressive effect on metastatic tumors, showcasing its comprehensiveness and efficacy in battling cancer diseases.

## 7 nMOFs for PDT + ferroptosis + CDT + GPX4 inhibitor

With unique porous structure, MOF nanomaterials exhibit exceptional drug-loading capabilities. This enables them to carry a diverse array of therapeutic molecules for complex treatment strategies (Wang Y.-M. et al., 2022; Liu et al., 2023). Specifically, porphyrin-based nMOFs serve as efficient carriers for the small-molecule inhibitor RSL3. RSL3 selectively targets and inhibits GPX4, a key antioxidant enzyme. GPX4 is responsible for scavenging lipid peroxides and maintaining cellular redox homeostasis (Chen et al., 2023b). Upon GPX4 inhibition, elevated levels of LPOs accumulate within the cell, thus triggering a lipid peroxidation cascade and generating excessive ROS. This ROS overproduction culminates in ferroptosis, an atypical form of cell death. Ferroptosis is iron-dependent and programmed, distinct from apoptosis, necrosis, or autophagy. After loading RSL3, porphyrin-based nMOFs can precisely modulate intracellular oxidative stress. This modulation efficiently induces ferroptosis, thereby showcasing substantial potential in cancer therapy (Chen et al., 2023b).

**FIGURE 7:**
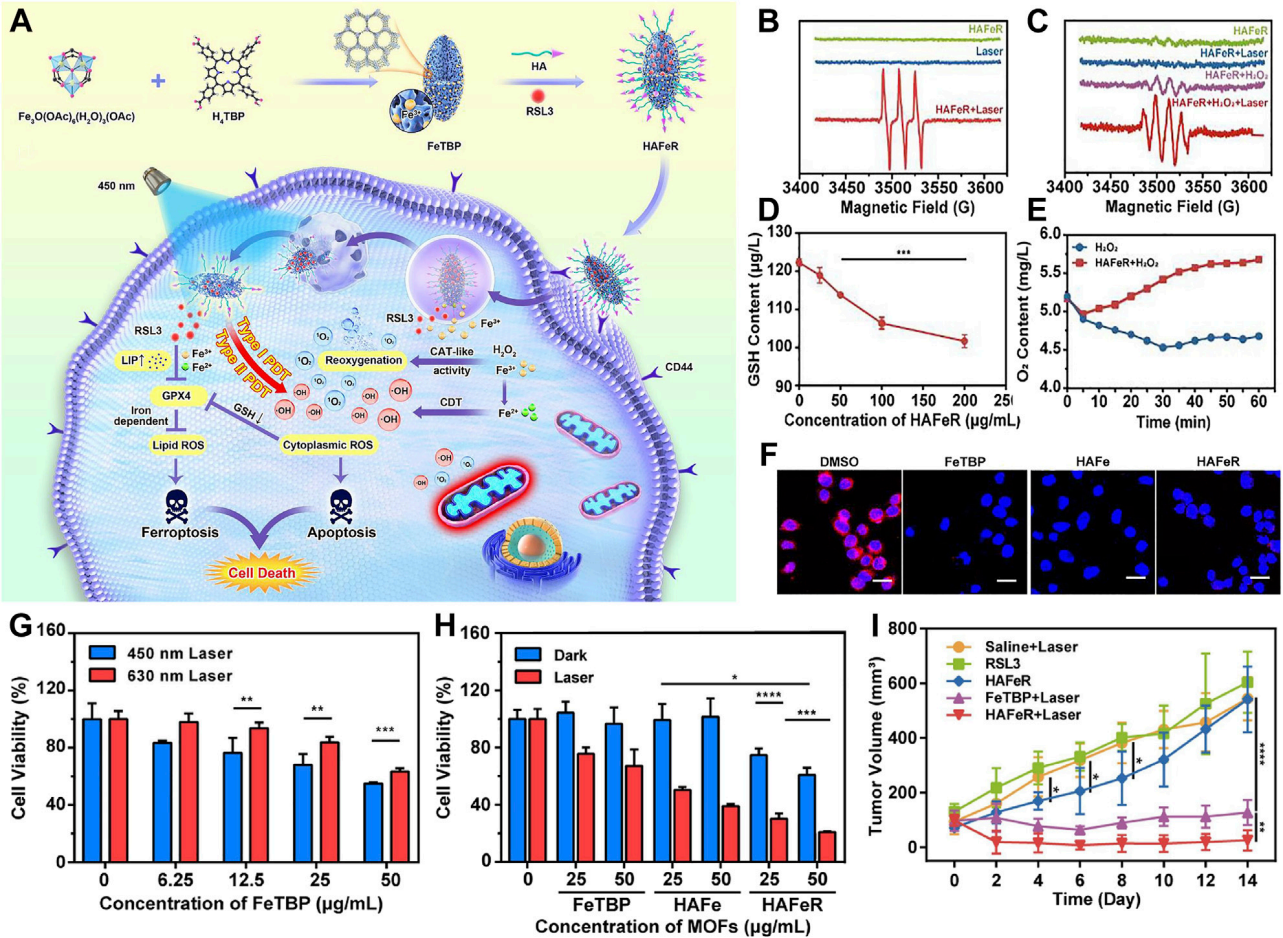
**(A)** Diagrammatic representation of HAFeR MOFs facilitating hypoxia-resistant and labile iron pool-enhanced synergistic ferroptosis and photodynamic therapy. **(B, C)** ESR spectral analysis of TEMP/^1^O_2_ **(B)** and DMPO/•OH **(C)** radicals generated by HAFeR (200 μg/mL) upon 450 nm laser exposure (30 mW/cm^2^, 15 min). **(D)** GSH depletion efficacy of HAFeR at varying concentrations post 450 nm laser irradiation (30 mW/cm^2^, 15 min). **(E)** Time course of O_2_ production by HAFeR, monitored by an oxygen sensor. **(F)** Intracellular O_2_ release detection using a hypoxia-sensitive probe (RDPP); scale bar = 25 μm. **(G)** Survival rate of 5,637 cells treated with FeTBP at different concentrations under 450 nm or 630 nm laser irradiation (30 mW/cm^2^, 10 min). **(H)** Viability of 5,637 cells exposed to FeTBP, HAFe, and HAFeR (25 or 50 μg/mL MOFs) followed by 450 nm laser irradiation (30 mW/cm^2^, 15 min); n = 3. **(I)** Comparative tumor growth profiles. Reproduced with permission from Chen et al. (2023b). Copyright (2023), Elsevier.

He et al. engineered a nanoparticle termed HAFeR, encapsulating a photosensitizer and RSL3 within a MOF, designed to concurrently trigger ferroptosis and PDT at the tumor site. By leveraging affinity for CD44 receptors, HAFeR specifically localizes and enters tumor cells. Under the acidic conditions of lysosomes, it subsequently releases porphyrins, iron ions, and RSL3. Upon excitation with 450 nm laser light, the porphyrins generate ROS with higher efficiency than 630 nm light, thereby initiating apoptosis. Catalyzed by iron ions, the Fenton reaction facilitates the conversion of H_2_O_2_ to O_2_, thereby ameliorating tumor hypoxia. Concurrently, RSL3 downregulates GPX4 thus accelerate lipid peroxidation and ultimately leading to ferroptosis. HAFeR demonstrates potent cytotoxicity against tumor cells (Figure 7A) (Chen et al., 2023b). ESR spectroscopy confirms the production of ROS by HAFeR upon 450 nm laser exposure, verifying the generation of ^1^O_2_ and hydroxyl radicals (Figures 7B, C). ROS detection assays confirm the sustained generation of toxic ROS by HAFeR under light exposure, exerting cytotoxic effects even under hypoxic conditions. HAFeR also significantly depletes GSH (Figure 7D), enhancing cellular sensitivity to ROS while producing O_2_ via H_2_O_2_ degradation (Figures 7E, F), improving oxygen supply in the tumor microenvironment. *In vitro* experiments demonstrate that HAFeR in combination with 450 nm laser light exhibits superior tumor cell killing compared to 630 nm light. Especially in the presence of RSL3, this combination achieves cell death rates exceeding 80%. This rate is markedly higher than using FeTBP or HAFe alone (Figures 7G, H). *In vivo* studies show that HAFeR in conjunction with 450 nm laser irradiation significantly inhibits tumor growth in MB49 bladder cancer-bearing mice. The suppression rate reaches as high as 95%. In contrast, mice treated with HAFeR alone display less pronounced tumor growth retardation (Figure 7I).

Through the synergistic action of exogenous iron ions and RSL3, this system could downregulate GPX4 expression. This downregulation potentiates the induction of ferroptosis. As a result, it enhances therapeutic efficacy. Being a pivotal enzyme in antioxidant defense, inhibition of GPX4 leads to the accumulation of lipid peroxides, accelerating the onset of iron-dependent cell death mechanisms. By combining apoptotic pathways with ferroptosis mechanisms, the HAFeR system exhibits formidable cytotoxicity against tumor cells. It not only selectively eradicates cancer cells but also clears transplanted MB49 bladder cancer cells more effectively under the 450 nm laser irradiation. This achievement reveals the integration of PDT with ferroptosis strategies as a novel direction in oncology. This integration promises an efficient and less toxic alternative to traditional chemotherapy. It is particularly promising for aggressive malignancies resistant to standard treatments.

## 8 NMOFs for PDT + ferroptosis + CDT + immunotherapy

Porphyrin-based nMOFs serve as carriers for GPX4 inhibitors. They can also deliver Oridonin (ORI), a natural terpenoid compound extensively studied as a novel anticancer agent. Delivering ORI improves the tumor microenvironment and disrupts intracellular iron homeostasis. This disruption augments ferroptosis therapy (Cai M. et al., 2024). ORI has been shown to induce ferroptosis in tumor cells by diminishing GPX4 activity and depleting intracellular GSH. Additionally, in combination with Calcium superoxide (CaO_2_), a compound that can ameliorate the hypoxic tumor microenvironment, this approach enhances therapeutic outcomes (Cai M. et al., 2024). CaO_2_ is considered highly biocompatible due to its sustained release of O_2_ and H_2_O_2_. Given its dual production of O_2_ and H_2_O_2_, CaO_2_ significantly amplifies the effects of PDT and CDT in treating hypoxic solid tumors. Moreover, the release of Ca^2+^ into the cell by CaO_2_ triggers calcium overload.

**FIGURE 8:**
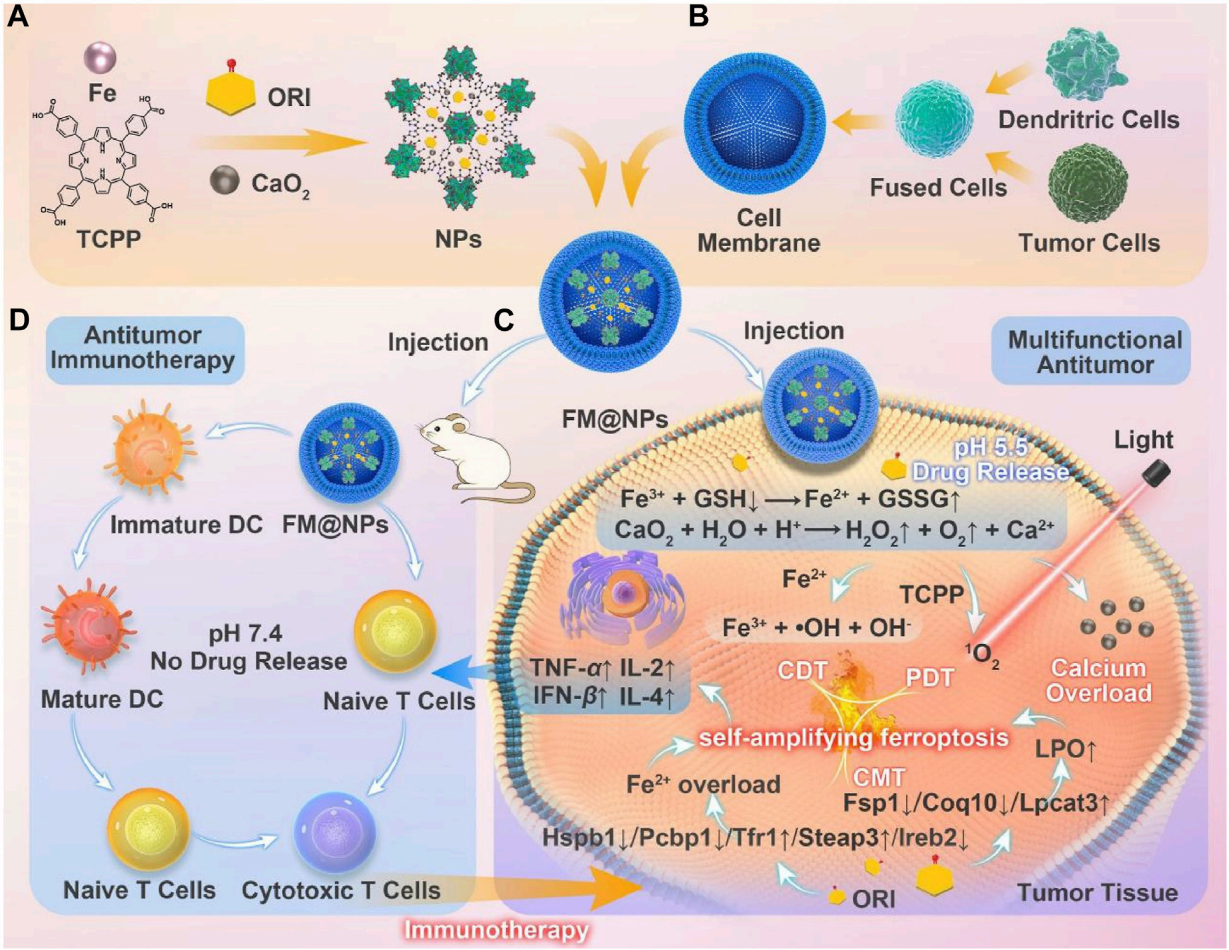
**(A)** Conceptual depiction of the fabrication process for FM@NPs derived from an iron-based MOF nanoplatform and **(B)** a hybrid cell membrane. **(C)** Enhanced ferroptosis therapy and PDT, facilitated by the engineered FM@NPs. **(D)** Augmented antitumor immunotherapy through the strategic design of FM@NPs. Reproduced with permission from Cai M. et al. (2024). Copyright (2024), Elsevier.

Building on these findings, Ni et al. developed a system utilizing an iron-induced ferroptosis nanoplatform of MOFs, incorporating iron supply and GSH depletion (Cai M. et al., 2024). Using a seed growth strategy, CaO_2_ was encapsulated within a nanoscale MOF layer (Fe-TCPP, FT) to prevent premature leakage of CaO_2_ into the bloodstream (Figure 8A). The nanoparticles (ORI@CaO_2_@Fe-TCPP, NPs) were further modified with a fusion membrane (FM) to create FM@ORI@CaO_2_@Fe-TCPP (FM@NPs) (Figures 8A, B) (Cai M. et al., 2024). The hybrid cell membrane was surface-modified to enable targeted immunotherapy. The entrapped ORI simultaneously suppresses the HSPB1/PCBP1/IREB2 and FSP1/COQ10 pathways, synergizing with Fe^3+^ to induce ferroptosis. Porphyrin-mediated PDT, characterized by the substantial accumulation of ROS, significantly enhances ferroptosis (Figures 8C, D). This self-amplifying strategy facilitates robust ferroptosis, which can synergize with fusion membrane-mediated immunotherapy.

This study proposes a biocompatible nanoparticle of CaO_2_, hybridized for growth with an iron-porphyrin MOF. The porosity of nMOFs is utilized to load triptolide, and a fused cell membrane coating enables synergistic effects of PDT, ferroptosis, and CDT. This approach enhances immunotherapy and successfully inhibits melanoma growth. This research validates the feasibility and promise of integrating immunotherapy, CDT, and efficient ferroptosis induction via nanotechnology as a viable strategy for melanoma treatment. The combined therapeutic modalities address distinct aspects of tumor biology. Each modality contributes to a comprehensive approach to overcoming the challenges posed by melanoma. This combination underscores the potential for multifaceted therapeutic interventions in clinical oncology.

## 9 Conclusion and perspectives

In recent decades, PDT has emerged as a pivotal modality in oncology, leveraging light-sensitive agents and targeted illumination to eradicate tumors with precision while sparing adjacent healthy tissue (Gunaydin et al., 2021; Zheng X. et al., 2021). However, clinical implementation has been hampered by limitations such as inadequate light penetration, uneven distribution of PSs, and tumor resistance to light exposure (Fan et al., 2016; Zhao et al., 2023). To overcome these hurdles, researchers are pioneering advancements in photosensitizer design, light source technology, and integrative therapeutic strategies, aiming to significantly bolster PDT’s efficacy and broaden its therapeutic horizon (Fan et al., 2016).

As a novel form of programmed cell death characterized by iron-dependent lipid peroxidation, ferroptosis has garnered attention for its potential to circumvent resistance mechanisms in cancer cells (Li et al., 2023; Upadhyayula et al., 2023; Wang et al., 2023b; Wu et al., 2023; Zeng et al., 2023). Despite its promise, challenges remain in efficiently inducing ferroptosis in tumors and mitigating the tumor microenvironment’s inhibitory effects on ferroptosis inducers (Mao et al., 2021; Wang C. et al., 2024). Innovative approaches are being pursued to advance the clinical applicability of ferroptosis. These approaches include refining ferroptosis inducer chemistry and exploring synergistic combinations with other therapies. Additionally, identifying biomarkers that enhance sensitivity to ferroptosis is another focus area (Battaglia et al., 2020; Tang et al., 2021).

Porphyrin-based nMOFs have emerged as versatile platforms for enhancing both PDT and ferroptosis, serving as efficient carriers for photosensitizers and facilitators of ferroptotic processes (Yang F. et al., 2023). This review highlights recent breakthroughs in the utilization of porphyrin-based nMOFs for synergistic PDT and ferroptosis-mediated cancer therapy, showcasing their potential for multimodal treatment strategies. By elucidating the interplay between PDT and ferroptosis within the nanomedical landscape, we try to uncover the vast potential of multifunctional carriers to orchestrate combinatorial therapeutic effects.

The development and application of porphyrin-based nMOFs herald a transformative era in clinical oncology. These nMOFs offer tailored, high-efficiency treatment regimens. Such regimens address tumor heterogeneity, multidrug resistance, and metastasis. The synergy between PDT-induced oxidative stress and ferroptosis provides a fresh perspective on overcoming therapeutic barriers. As our understanding of the mechanistic underpinnings of porphyrin-based nMOFs in PDT and ferroptosis deepens, and with ongoing innovation in multifunctional carrier design, these advanced materials are poised to revolutionize cancer therapy (Wu J. et al., 2024).

From our perspective, the efficacy of porphyrin-based nMOF-mediated PDT combined with ferroptosis can be enhanced in several ways. Despite extensive research on porphyrins, their maximum absorption wavelength is approximately 640 nm. This wavelength is associated with a relatively low molar extinction coefficient, which is not ideal for light penetration through tissue or the excitation of photosensitive materials. Utilizing reduced derivatives such as chlorins or bacteriochlorins could be a promising alternative. Chlorins exhibit a molar extinction coefficient at ∼640 nm that is 10 times greater than that of porphyrins, facilitating more efficient photon capture (Lu et al., 2015). Bacteriochlorins have their peak absorption shifted to around 740 nm (Luo et al., 2020). This shift places them within the near-infrared window. As a result, the penetration of the excitation light source through tissue is enhanced. Secondly, employing sonodynamic therapy or X-ray excitation for porphyrin PSs might yield unexpected therapeutic benefits (Yang et al., 2021). Once again, as the cellular organelles responsible for energy supply, mitochondria play a critical role in maintaining normal cellular metabolism (Xu et al., 2020; Yang et al., 2020; Wang et al., 2021). Research targeting these organelles has become a pivotal direction in elucidating the mechanisms of various diseases (He-Yang et al., 2020; Cheng et al., 2021; Song et al., 2021). We posit that designing porphyrin-based nMOFs targeted at mitochondria may yield unexpected and superior therapeutic outcomes when applied in the combined treatment of PDT and ferroptosis. Lastly, integrating theranostics into cancer treatment is essential. Leveraging the imaging capabilities of the metallic components within the nMOF framework allows for real-time monitoring of drug distribution across various tissues and organs, thereby optimizing treatment efficacy while minimizing drug concentration.

Accelerated interdisciplinary collaboration and clinical trials will further substantiate the clinical utility of porphyrin-based nMOFs, hastening their translation into effective treatments for cancer patients. The future of cancer care is brightened by the prospect of harnessing these cutting-edge materials to usher in a new era of personalized and potent therapeutic interventions.

## Abbreviations

PDT, photodynamic therapy; nMOF, nanoscale metal organic framework; PSs, photosensitizers; ROS, reactive oxygen species; LPO, lipid peroxidation; GSH, glutathione; GPX4, glutathione peroxidase 4; CDT, chemodynamic therapy; Ce6, chlorin e6; NP, nanoparticle; RMOF, redox-responsive MOF; HA, hyaluronic acid; ESR, electron spin resonance; MTT, 3-(4,5-Dimethylthiazol-2-yl)-2,5-diphenyltetrazolium bromide; CO, carbon monoxide; NO, nitric oxide; TK, thioether; TPP, triphenylphosphine; TPZ, tirapazamine; ORI, oridonin.
